# Evaluating Improvement Collaboratives in Quality Improvement Projects: Design Variations and Their Impact

**DOI:** 10.1192/bjo.2024.417

**Published:** 2024-08-01

**Authors:** Geetika Singh, Mehtab Ghazi Rahman, Isaac Obeng, Lucy Palmer, Janet Seale

**Affiliations:** Central and North West London NHS Foundation Trust, London, United Kingdom

## Abstract

**Aims:**

Aim: To compare and evaluate three improvement collaboratives designs in terms of tangible and non-tangible benefits.

Background: Leading health systems have invested in substantial quality improvement capacity building, but little is known about the aggregate effect of these investments at the health system level.

Collaborative learning is one of the educational approaches of using groups to enhance learning through working together. Research shows that collaborative experiences that are active, social, contextual, engaging and student-owned leads to deeper learning.

**Methods:**

CNWL organised three collaborative programmes with varying duration and distinct approaches to team selection, wrap-around support mechanisms, training design and post-collaborative QI support.

These three virtual collaborative programmes were co-designed with service users and carers to support 24 teams each in planning, delivering and sustaining improvements aligned with the Trust's Strategic Priorities.

All programmes provided knowledge on the Model for Improvement and co-production, enabling frontline ownership of safety solutions while building organisational QI capacity and capability.

Each collaborative was divided into Planning and Delivery phases. The evaluation, which covers a 3-year period, compares programme metrics to assess effectiveness, impact and identify areas for improvement.

**Results:**

Incorporating cognitive diversity is crucial in improving the learning process. Collaboratives play a vital role in achieving this, as they bring together different services, staff, and SU&C to drive improvement.

The benefits of collaborative work in quality improvement extend beyond the project data, as it can lead to positive unintended consequences such as a shift in team culture and the adoption of an improvement mindset. These outcomes gained on the journey should be evaluated and celebrated. Moreover, collaboration fosters a culture and platform for sharing and spreading learning beyond the team/service.

However, it is important to take the time to consider and compare different designs of collaboratives during the scoping phase. Factors such as the duration of the collaborative programme, the need for additional wrap-around support and the selection of measures to evaluate the programme should be carefully considered before proceeding.

Effects of changes

1. Comparing different collaborative designs identified the key enablers to a successful project. They were application process brought teams together that were ready and willing to improve; targeted wrap-around support to Sponsors, SU&Cs, Coaches and having decision gateways in design enabled focused and candid conversations about team progression.
2. Collaborative with longer time frame were more resource intensive but had a greater positive impact on safety culture, successful projects and sustained gains than the shorter duration.
3. CNWL Added Value framework evaluated tangible and non-tangible benefits, i.e. staff experience, safety and learning culture, patient experience, streamlined processes and efficiencies gained.

**Conclusion:**

It is important to look at the local context when designing a collaborative with their clinical setting.

A consideration should be based on resources available to support the entire duration of collaborative and what are the desired outcomes of the collaborative.

